# Learning for life, friendships and relationships from the perspective of children and young people with intellectual disabilities: findings from a UK wide qualitative study

**DOI:** 10.1186/s12889-024-19972-y

**Published:** 2024-09-12

**Authors:** Michael Brown, Mark Linden, Lynne Marsh, Maria Truesdale, Fintan Sheerin, Freda McCormick

**Affiliations:** 1 School of Nursing and Midwifery, Queen’s University Belfast, 97 Lisburn Road, Belfast, BT9 7BL UK; 2 Scottish Learning Disability Observatory, University of Glasgow, Glasgow, G12 0XH UK; 3 School of Nursing and Midwifery, Trinity College Dublin, Dublin, D02 PN40 Ireland

**Keywords:** Children, Health, Inclusive education, Intellectual disabilities, Pupils, Qualitative research, Relationships, Sexuality, Special schools, Young people

## Abstract

**Background:**

Relationships and sexuality education (RSE) programmes are widely taught in schools, however for children and young people with intellectual disabilities, these programmes appear to be limited regarding information on relationships, informed choices and decision making. The purpose of this study was to seek the views and understanding of children and young people with intellectual disabilities, and those involved in their care and education, to identify best practice and approaches to the delivery on relationships and sexuality education.

**Methods:**

This study used a qualitative design with 37 pupils from five special schools from across the United Kingdom (UK) participating. In-depth semi-structured interviews were held online, or in person. All interviews were recorded and transcribed verbatim. Transcripts were anonymised, assigned a pseudonym and subjected to inductive thematic analysis.

**Findings:**

Four themes emerged from the data: (i) enthusiasm and inquisitiveness to acquire knowledge; (ii) dynamics of positive friendships; (iii) experiences and understanding of supportive relationships and sexuality; and (iv) valuing the exchange of knowledge and information. The findings highlight that children and young people with intellectual disabilities want education, support and information on matters relating to their relationships and sexuality.

**Conclusions:**

This is the largest study to date providing a voice to children and young people with intellectual disabilities regarding their relationships and sexuality. While special schools provide relationships and sexuality education, there is a requirement for a programme and resources specific to the needs of pupils with intellectual disabilities to be developed and evaluated. Such education should continue beyond school and be embedded in adult services.

**Supplementary Information:**

The online version contains supplementary material available at 10.1186/s12889-024-19972-y.

## Background

Relationships and Sexuality Education (RSE) programmes are widely taught in schools. All UK schools are required to offer accessible and inclusive RSE to all pupils, including those with special educational needs and disabilities (SEND) [1–5].

The aim of RSE is to provide children and young people the information they need to help develop healthy, nurturing relationships of all kinds, including intimate ones. According to Barbagallo and Boon [6], previous RSE programmes focused on human reproduction and sexual organs [7], and lacked the teaching of emotions, relationships, and sexual health and well-being. However, more recently RSE has been considered a lifelong process of acquiring information, building respect for self and for others, and forming attitudes and ideologies about topics such as identity, relationships, reproduction, sexual health, and intimacy [8].

The past several decades has seen increased attention on the sexual rights and needs of people with intellectual disabilities, and their importance has been highlighted in several international policy reports [9–11]. However, despite the rights to RSE for all children and young people with intellectual disabilities being enshrined in the United Nations Convention on the Rights of the Child, (Article 25a) [12], evidence-based and rigorously tested RSE programmes appear to be absent.

There are challenges faced by pupils with intellectual disabilities in accessing comprehensive RSE information. This is attributed to some teachers omitting sexuality related content in RSE programmes and teaching delivery and lack of accessible resources [13, 14]. Studies have identified that young people and adults with intellectual disabilities are not provided with adequate relationships and sexuality education to prepare them for future relationships, sex and parenthood [13, 15]. Furthermore, many do not receive sexuality education that is orientated to their needs and development, promotes a positive image of sexuality, or enables and supports informed decision making [16]. Evidence across a 20-year study showed that children and young people with intellectual disabilities continue to be less likely to receive sex education [17, 18], notably those with profound and multiple intellectual and multiple disabilities. Carter et al. [13] assert that exclusion from RSE can have consequences for the sexual and reproductive health and well-being of people with intellectual disabilities. While the reticence to provide appropriate RSE is often rationalised with the intention of protecting vulnerable children and young people with intellectual disabilities, this may leave them more vulnerable to the potential of sexual violence, harm and exploitation [19].

The needs of children and young people with intellectual disabilities concerning sexuality and privacy are like their typically developing peers without intellectual disabilities with research evidencing that they find RSE helpful in addressing their needs [16, 20, 21]. Moreover, children and young people with intellectual disabilities may not have had the experiences to develop social skills for long-term relationships, even though they place a high priority on having friends and a desire to be more knowledgeable about sexuality [22, 23]. Children and young people with intellectual disabilities have the same desires regarding sexuality and reproduction as those without intellectual disabilities yet some continue to face restrictions in making choices about their bodies and developing friendships and intimate relationships [24–27].

Parents and teachers however may withhold information from children and young people with intellectual disabilities, to shield them from knowledge of the potential harms and threats that may exist in society [28, 29]. In schools, there are few RSE curricula specifically adapted for pupils with intellectual disabilities, with little evidence regarding the effectiveness, with notable exceptions using peer education approaches [25, 30]. Education regarding sex, particularly in the context of intellectual disability, appears to depend on what teachers are knowledgeable and comfortable delivering [28, 29]. The format of current RSE programmes have not been uniformly developed or evaluated. Brown et al., [31] identified the need to include people with intellectual disabilities in the planning phase of an education programme to ensure their needs and concerns are included and addressed. While adapted RSE for children and young people with intellectual disabilities is relevant and necessary, the wider knowledge and understanding about their specific views and perceptions remains limited. This may be attributed to the challenges of conducting such studies, as the range and presentation of intellectual disabilities may potentially impact on the ability of some to consent and participate in research studies.

The aim of this study was to explore the views and experiences of children and young people with intellectual disabilities, parents and professionals in the provision of RSE programmes in special schools across the UK. Due to the scope and extent of the wider dataset this paper provides a more detailed analysis of findings from the main data [32]. Data specific to the voice of pupils with intellectual disabilities were extracted for furthermore detailed analysis and are presented in this paper.

## Methods

### Design

A qualitative design was utilised for this study involving in-depth semi-structured and focus group interviews. An easy read information sheet and consent form were prepared for pupils with intellectual disabilities. The interviews and focus groups provided an in-depth understanding of pupils with intellectual disabilities’ experiences of relationships and sexuality education. Ethical approval was gained from an institutional review board at the lead author’s University (Ref: MHLS 19_19).

### Participants

Five special schools across England (*n* = 1), Northern Ireland (*n* = 2), Scotland (*n* = 1), and Wales (*n* = 1) took part in the study. Special schools accommodate pupils whose educational needs cannot be met within mainstream schools. The focus is on the pupil’s individual needs and learning styles with appropriate teaching support and additional therapies and supports provided. Class sizes are smaller comprising pupils with similar needs rather than age [33]. Potential pupil participation in the study within each school was determined by a gatekeeper, who was the principal or a designated teacher. The gatekeeper knew the pupils and was best placed to judge their capacity to consent and likely willingness to participate. A total of 37 pupils participated from England (*n* = 5), Northern Ireland (*n* = 24), Scotland (*n* = 5) and Wales (*n* = 3). The age of pupils ranged from 11 to 19 years, and all were able to understand and articulate responses to the research questions. The demographic details of the pupils are shown in Table 1.

**Table 1:** Demographic details of pupils

Characteristics	Category	Number	(%)
Gender	Male	23	(6)
	Female	14	(38)
Age	11	1	(3)
	12	1	(3)
	13	2	(5)
	14	3	(8)
	15	4	(11)
	16	2	(5)
	17	2	(5)
	18	1	(3)
	19	1	(3)
	Boys group 13–19	12	(32)
	Girls group 12–19	8	(22)
Country	England	5	(13)
	Northern Ireland	24	(65)
	Scotland	5	(14)
	Wales	3	(8)

### Data collection

A gatekeeper in each school identified pupils willing to participate in an interview or focus group and issued letters of invitation and information about the study to the children and their parents. The researcher liaised with each gatekeeper to arrange interviews with their consent. Prior to participation all pupils were required to complete an accessible consent form, with a parent also providing written assent. They were informed of the right to withdraw from the study at any time, without giving any reason; limits of confidentiality and data protection; and an option to being contacted and invited to take part in future studies of a similar nature with no obligation.

The research team developed an interview guide that included probing questions and prompts for the study (see supplementary material 1). The researcher used this guide in all individual and focus group interviews to ensure consistency. Interview questions were open-ended to encourage participants to provide detailed responses regarding experiences of RSE programmes, and understanding of relationships, sexuality and consent. Examples of good practice, methods of delivery and topics for inclusion in an RSE programme were also explored.

Individual interviews took place with 13 pupils via Microsoft Teams, and 4 pupils face-to-face. Interviews lasted between 10 and 22 min. The remaining 20 pupils took part in two focus groups from one school consisting of an all-boys or all-girls group. The boys’ focus group had a total of 12 participants and lasted 34 min; while the girls’ focus group had a total of 8 participants and lasted 39 min. Each focus group, and some interviews, had a member of staff present for additional support.

All individual and focus group interviews were recorded and transcribed verbatim. Transcripts were anonymised by removing all identifiable information and assigning each participant a pseudonym.

### Data analysis

The six phase approach by Braun and Clarke [34] was used to guide data analysis. First, the research team independently read the transcripts to gain an understanding of the participants’ experiences. Next the transcripts were thematically analysed by each member of the research team to identify key themes. Following this, the transcripts were discussed as a team to identify and agree the final themes [34]. NVivo 12 [35], a data management programme, was used to facilitate analysis and management of the data. The approaches to data collection and analysis were rigorously followed by the research team to ensure the credibility, trustworthiness and dependability of the process which included investigator triangulation, audit trail of interviews, and thick, detailed description [36, 37].

### Rigour

In addition to the application for ethics approval, a research protocol was prepared and peer [34] reviewed. To ensure consistency across interviews and focus groups the same researcher (FM) conducted all data collection using an interview guide and transcribed recordings. This ensured accurate transcripts guaranteeing precise representation of participants’ perspectives. The researcher had extensive experience of conducting interviews and focus groups involving people with intellectual disabilities. The data analysis attained credibility through meticulous validation by all members of the research team.

## Findings

Four themes emerged following analysis of the data: (i) enthusiasm and inquisitiveness to acquire knowledge; (ii) dynamics of positive friendships; (iii) experiences and understanding of supportive relationships and sexuality; and (iv) valuing the exchange of knowledge and information.

### Enthusiasm and inquisitiveness to acquire knowledge

There was enthusiasm amongst all the pupils with intellectual disabilities to learn about RSE, and they also wanted the opportunity to extend their learning beyond the school years. This yearning for knowledge ranged from understanding body parts, friendships, puberty, and how to form relationships, to how to have a baby and not just how to prevent such an occurrence. There were many different modes of delivering such information, both in the classroom setting and at home from family members such as parents and grandparents. However, irrespective of the educator, it was important to the pupils that the “truth” (Pupil 31, age 14) and clear information was given. Whilst some pupils were not allowed access to social media, or had removed themselves due to negative experiences, others were using social media and accessing the internet for information. RSE was considered as advantageous amongst the pupils, with one stating they had learned “to be safe and watch out, and take care of yourself” (Pupil 34, age 15). Nevertheless, one pupil came to the realisation that the internet “is a very dangerous place” (Pupil 25, age 12–19) with others being aware of the importance of following guidance from parents and other family members to keep them safe.

*I think even after school as well. Like it’s always expanding*, *like relationships and things and there’s always like new things to learn about with it. (Pupil 12*, *age 18)*

*I get my information from online*, *Mrs [teacher]*, *or anyone else willing to teach me … Some of it I have self-teached myself and others it’s from teachers. I sort of give it variety. Some of it I learn and self-learn and others I learn from other people. (Pupil 2*, *age 15)*

*I have been learning it [RSE] with my parents. My parents have been privately talking about it as well … what I found is*, *my parents tell me the right way about relationships and the right way because some people might say it the other way and it can’t be true. (Pupil 25*, *age 12–19)*

*How they talk about consent*, *it’s very good*, *yes or no. Not maybes*, *just yes and no. And they talked about the ages what was very good*, *I think. And how you can get in a lot of trouble. (Pupil 33*, *age 15)*

*I feel safe because I have had plenty of guidance. So*, *my sister is connected to me. And my mum looks at my phone all the time. So*, *I feel safe. (Pupil 25*, *age 12–19)*

#### Dynamics of positive friendships

The dynamics of having or being a friend were well articulated by the pupils with descriptive words such as “nice”, “helpful” and “polite”. The importance of having a supportive friend in terms of listening and talking about problems was recognised by some as valuable and much needed. This was characterised with the need for the person to be honest and trustworthy. Friendships were predominately forged in school with few of the pupils having friends in other settings such as sports clubs and their local neighbourhood. The use of social media platforms was prevalent amongst the pupils with many feeling safe while using them. However, this was primarily for keeping in touch with both their school friends and family members. Irrespective of where they liaised with their friends, the pupils were mindful of the importance of treating each other well and having respect. Where these values were compromised, the pupils had no hesitation terminating friendships or blocking contact online.

*Somebody you can trust. Somebody you can see every now and then. Being there for you. Sometimes I listen to their problems. I calm their nerves sometimes if they are really nervous. I tell them all it’s going to be ok. (Pupil 31*, *age 14)*

*I have only got friends what are in school what I play with on Xbox and games. That’s where we chat. (Pupil 3*, *age 13)*

*The only difference between a friendship online and offline is online you don’t really see much of their face or where they are. Which is why I often tend to say if you want me to be your friend you need to meet me in person. We need to meet in person so I know you better … And if they keep trying it*, *I just block them and if they continue to try it*, *I just report them. (Pupil 2*, *age 15)*

*You don’t want someone that will put you in a bad path and get you in trouble. (Pupil 28*, *age 12–19)*

### Experiences and understanding of supportive relationships and sexuality

There was mixed understanding amongst the pupils on the concepts of friendships, relationships, sexuality and sex. When asked about sexuality some pupils responded they were “not quite sure” or “don’t really know”, whilst for others, they were aware and knowledgeable of gender diversity. A few pupils shared personal experience of being transgender, gay or bi-sexual. Consent was generally understood amongst the pupils with reference to “permission” often used. The importance of respecting a person’s response and the consequences of not doing so was appreciated and understood by most. Furthermore, the concept of knowing “what’s right and wrong” featured across the narratives. This was not only in terms of consent but also regarding being vulnerable and the prevalence of abuse in relationships.

*Well that is how you pretty much bring babies into the world. Create new people. (Pupil 3*, *age 13)*

*It’s practically just like*, *relationship wise and well*, *just like*, *like*, *it’s how to explain*, *but like*, *having a girlfriend*, *or a boyfriend. What I learned was like sexuality*, *like who you like too*, *like boys or girls and maybe trans*, *like all that*, *if you understand me. (Pupil 33*, *age 15)*

*Just making sure yes is yes*, *and no is no. So*, *yes is they want to do it*, *and no is*, *don’t be touching them*, *don’t be doing anything. Just have common sense. (Pupil 32*, *age 14)*

*It is important to know how a person will react and what they are used to and what they are not comfortable with. It is just important to know all this stuff because if you don’t*, *it could go wrong*, *very wrong. (Pupil 2*, *age 15)*

*Sometimes in relationships you need to find the signs of red flags when abusive relationships and then you go to someone and tell them what your partner or friend or like anyone that’s abusing you*, *or like children too. (Pupil 28*, *age 12–19)*

### Valuing the exchange of knowledge and information

It was very clear that the exchange of information relating to relationships and sexuality education from the educators and between their peers was appreciated by all. Whilst different modes of learning took place, classroom-based practical learning with teachers was the perceived preference amongst the pupils. For some, there was an element of embarrassment regarding some words and topics used, or at the thought of parents being involved. In general, however all pupils were prepared to talk openly and have discussions amongst their peers and with teaching staff. The value of the information being imparted was deemed important by the pupils as it was relevant to all stages of their lives from puberty to long term relationships, and associated legislation.

*It’s important to learn because if you don’t know then you don’t know what’s happening to your body. (Pupil 23*, *age 12–19)*

*I’m bi so I like girls and boys. Yeah*, *so*, *it like helps me know what I like and what I am attracted to. (Pupil 28*, *age 12–19)*

*It is just interesting learning about sex education … it is just great to know all this stuff because it can come in useful. (Pupil 2*, *age 15)*

*I think it’s very useful for life and going forward in relationships like what you are going to do when you’re older and your own choices … I reckon it’s so important because like*, *it can be too late and then a baby comes. Then practically your childhood’s ruined because you have a baby and all your friends are going out to clubs or whatever*, *and you’re sitting in the house minding a baby. (Pupil 33*, *age 15)*

## Discussion

Our findings provide new insights and understanding of how children and young people with intellectual disabilities have experienced relationships and sexuality education. It has evidenced that they want to know about the topic and see the value in learning about relationships and sexuality. Participants were enthusiastic and curious to learn more and were cognisant of the possible dangers around seeking information from the internet or social media. A systematic review on how people with disabilities used social media identified six themes including community, cyberbullying, self-esteem, self-determination, access to technology and accessibility [38]. Community was the largest reported theme and referred to people coming together to share common interests, needs, ideas or for the purpose of collaboration, support and identity development [38]. People with intellectual disabilities routinely utilised social media and social networking sites to stay in touch with friends, enjoying the experience. However, some have experienced bullying and exploitation [39]. Pupils in our study sought information about relationships and sexuality from both online and offline sources. Therefore, it is important that information provided is accurate, accessible and free of judgement to ensure children and young people remain safe and well informed.

The pupils valued positive friendships and sought peers who held similar interests. Attributes such as trustworthiness, politeness, being nice and helpful were all seen as important. As with their typically developing peers, children and young people with intellectual disabilities desire belonging and friendship which often comes with a certain degree of conflict [40]. Our participants also made use of social media to keep in touch with school friends and family and were not averse to blocking contact if they were treated poorly. Friendships can provide emotional support, increases self-worth and well-being and are an important source of information [41]. In some cases, friendships among children and young people with intellectual disabilities can be difficult to maintain and develop and can be damaged by prejudice on the part of peers and others [42]. Therefore, RSE should provide information to children and young people with intellectual disabilities on how to negotiate, navigate, maintain and terminate friendships and how to identify the characteristics of good and poor relationships.

The concept of sexuality elicited a variety of responses from our participants. Some pupils were aware of the diverse expression of sexuality while others were unsure what this referred to. Research evidences that adults with intellectual disabilities want access to information about expression of their sexuality, rights and responsibilities, including protection from exploitation and abuse [15]. As adults with intellectual disabilities have identified this need, current educational provision is clearly inadequate leaving children and young people at greater risk of harm, exploitation and sexual abuse [28]. It is therefore important to have an open and honest discussion with children and young people about their sexuality to ensure they are well informed and can understand differences in the expression of human sexuality safely. This is particularly important in cases where children and young people with intellectual disabilities do not identify as heterosexual. There is currently limited support for people with intellectual disabilities who identify as lesbian, gay, bi-sexual or transgender [15, 43]. It was encouraging to identify that our participants recognise the concepts of consent and respect. This is positive, as research has evidenced that some adults with intellectual disabilities scored lower on a measure of consent and abuse than typically developing adolescents [44]. However, the authors suggest capacity to consent may undergo change and can be dependent on the young person’s level of knowledge and situation [44]. The development of an RSE programme would have to place issues of consent to sexual intimacy at the centre and should seek to increase the knowledge of children and young people to support healthy and safe sexual decision-making and experiences.

The pupils in this study preferred to receive their information from teachers and saw them as a trustworthy source. While peers and parents were also discussed as sources of learning these interactions were thought to be less reliable and potentially more awkward or embarrassing. Delivery of RSE in special schools has experienced some challenges such as a lack of training for teachers and inadequate materials for use with pupils with intellectual disabilities [28]. While programmes have been developed for typically developing children and young people, no evidence-based programme has yet been developed specifically for the intellectually disabled pupils. Research has identified that people with intellectual disabilities want information about sexual health and have often had to acquire this knowledge through lived experience [45]. Delivering an evidence-based RSE resource at school is the preferred method of learning for our participants. However, it is important to keep parents and carers informed and apprised of RSE content delivery to ensure any conversations at home did not come as a surprise and any misunderstandings could be sensitively addressed.

### Policy implications

Children and young people with intellectual disabilities have the right to education under Article 24 of the United Nations Convention on the Rights of Persons with Disabilities [10]. Article 24 states that people with disabilities should be enabled to learn life and social development skills to facilitate equal participation in education and their communities [10]. As such, the state has a legal requirement to provide education which meets the needs of people, including children, with intellectual disabilities. To support children and young people with intellectual disabilities to participate in their communities, educational policy makers should ensure the provision of good quality RSE which meets their needs, is evidence-based and rigorously tested. Not only should RSE be available to pupils with intellectual disabilities, in addition, content and teaching needs to be tailored to meet specific learning needs taking account of their developmental stages [3].

Our findings support this position and evidences that children and young people with intellectual disabilities want to learn about relationships and sexuality and see the benefit of acquiring this information. To enable this, there is a need to create and implement evidence-based programmes for pupils with intellectual disabilities in schools. Such programmes would have the benefit of being directly relevant to the needs and concerns of pupils and would address, in an easily accessible manner, RSE issues that affect them. Education policy makers should ensure that programmes are rigorously developed, tested, implemented and evaluated to allow children and young people with intellectual disabilities access to this important information.

RSE policy guidance is available for typically developing children and young people and is required for those with intellectual disabilities. Policy makers should co-design and co-produce RSE guidance with the range of key stakeholders including children and young people to ensure the development of a fully compressive strategy. To be effective, RSE policy needs to set out the delivery context of RSE for children and young people with intellectual disabilities, taking account of age, set within cognitive ability level. RSE policy specific to the needs and concerns of children and young people with intellectual disabilities clearly setting out the broad areas that should be the focus of programmes to ensure consistency and allow for the impact and outcomes to be identified.

The current research has focused on pupils with intellectual disabilities in a special school setting. The findings and recommendations arising from the study offers a way forward to grow and build on the work currently taking place regarding RSE. It is therefore relevant to policy makers, professionals working with children and young people with intellectual disabilities in school settings and their families. The inclusion and participation of the parents and families of children and young people with intellectual disabilities are also key to the effective development and delivery of RSE. Through their participation in the current study, it is evident that children and young people with intellectual disabilities can and want to be included as the key consumers of RSE.

This is important as all children and young people, including children and young adults and adults with intellectual disabilities require information regarding relationships and sexuality. RSE information will differ to that provided to school aged children, however it applies equally across the lifespan and is lifelong. Therefore, appropriate educational provision regarding RSE should continue through day services to support and help ensure young adults with intellectual disabilities develop safe and fulfilling friendships and relationships after they leave school. This approach could also benefit older adults with intellectual disabilities who may not have received any tailored evidence-based RSE while at school specific to their needs and ability level.

### Strengths and limitations

To our knowledge, this is the largest study to identify the voices of children and young people with intellectual disabilities regarding RSE. Many studies to date have focused on the perspectives of families and carers, adults with intellectual disabilities and professionals, omitting the views of children and young people. Therefore, the findings of the study provide an in-depth understanding of the experiences of pupils with intellectual disabilities in special school settings regarding RSE provision. Further, this study presents the views of pupils from across the UK and is not therefore limited to one single geographic region. This has increased the representativeness of our findings. However, many participants came from Northern Ireland, and no representation of non-verbal children and young people, which may have influenced the results and wider relevance. Our use of both semi-structured interviews and focus groups may also have influenced the findings. While there were practical considerations in using both approaches, for example, a focus group took up less classroom time, the different dynamics involved (group discussion versus one-to-one discussion) may have resulted in less detailed narratives from pupils involved in focus group interviews. This study did not include information of the level or grading of intellectual disability and therefore our findings may not be generalised more widely. Implementation of RSE curriculum requires careful planning to make it accessible for specific types and level of intellectual disability.

## Conclusions

The findings from this study highlight the views and experiences of pupils with intellectual disabilities with regard to their learning about RSE. Undoubtedly, these children and young people want information about relationships, sexuality and sex, to increase their understanding and knowledge to help protect them as they move into adulthood and leave school. While special schools provide relationships and sexuality education, there is a requirement for a programme and resources specific to the needs of children and young people with intellectual disabilities to be developed and evaluated. Furthermore, the content requires to be personalised and tailored to their developmental stages. In addition, a programme should be developed to enable RSE to continue beyond school and be embedded in adult services.

## Electronic supplementary material

Below is the link to the electronic supplementary material.

Supplementary Material 1: Interview guide

## Data Availability

The data that support the findings of this study are not openly available and may be available from the corresponding author upon reasonable request.

## Abbreviations

RSE: Relationships and sexuality education
SEND: Special educational needs and disabilities
UK: United Kingdom
