# Supplementary material for: Learning for life, friendships and relationships from the perspective of children and young people with intellectual disabilities: findings from a UK wide qualitative study
Source: BMC Public Health. 2024 Sep 12;24:2491. doi: 10.1186/s12889-024-19972-y (PMC11396106; doi:10.1186/s12889-024-19972-y)
Supplement: Supplementary file 1 — Supplementary Material 1: Interview guide [file 12889_2024_19972_MOESM1_ESM.pdf]

Interview Guide for Pupils

Introduction

- Thank you for taking part in our research study and completing your consent form. Although you have given permission to take part, you are free to withdraw at any point during the interview or at any other time.

- I would remind you that our chat is being recorded and should last about 30 minutes to an hour depending on your answers. If you need to take a break or anything in between times just let me know.

- This study is about relationships and sexuality education for young people with learning disabilities. We would like to find out what you would like to learn about relationships. These could be about friendships, having a boyfriend or girlfriend, or could be about sex. It is important that we hear from young people on these issues so that we can tell adults who work with you about what you need.

- Are there any questions you would like to ask before we start?

1 Can you tell me a wee bit about you and your family?

2 What do you like to do with your friends in School and at home?

3 What does having or being a friend mean to you?
    - Having someone to do things with, connection, bond, closeness
    - Someone to talk to and share things

4 Sexuality is about your sexual feelings, thoughts, attractions and behaviours towards other people. It is not about who you have sex with, or how often you have it. Sexuality is diverse and personal, and it is an important part of who you are.

- What does sexuality mean to you?
    - Having a girlfriend or boyfriend
    - Dressing how you want
    - Having sex/intimate relationships

5 What makes a good friend or relationship, including those we have online?
    - Trust, respect, honesty, kindness, generosity, boundaries, privacy, consent
    - Management of conflict, reconciliation, ending relationships

6 Do you use any online platforms on your computer or phone to chat or interact with your friends?

- How safe do you feel when doing this?

- Tell me about any experiences you have had.

- Is there anything else we need to remember with our online friends and relationships?

    - Rights of everyone, safety, responsibilities, and opportunities
    - Risks, material shared online, difficulty removing potentially compromising material

7 Can you tell me what you know about consent?
    - Give and withdraw consent, including sexual consent
    - Recognise consent giving and withdrawal from others, including sexual consent
    - Recognise the characteristics and positive aspects of healthy one-to-one intimate relationships, which include mutual respect, consent, loyalty, trust, shared interests and outlook, sex and friendship

8 How have you learned about romantic relationships and sexuality?
    - Family, siblings, friends, teacher, internet, etc
    - RSE programme in school

- What did you find useful about this?

9 What would you like your parents and teachers to tell you about relationships and sexuality?
    - Respectful relationships including friendships
    - Children’s online safety and relationships
    - Positive sexual health, rights, safety, resources, etc.
    - Intimate and sexual relationships including sexual health

10 What would be the best way for you to learn about relationships and sexuality?
    - video/talk with parents/teachers, peer discussions, individual, etc.

11 How important do you think it is to learn about relationships and sexuality in school?

12 Are there any other comments you would like to make about what we have discussed?

- That’s all of my questions. Have you any questions that you would like to ask me?

- Thank you again for your time and taking part in our research.
